# Effect of Cr(V) on reproductive organ morphology and sperm parameters: An experimental study in mice

**DOI:** 10.1186/1476-069X-4-9

**Published:** 2005-05-27

**Authors:** Maria de Lourdes Pereira, Ricardo Pires das Neves, Helena Oliveira, Teresa Margarida Santos, Júlio Pedrosa de Jesus

**Affiliations:** 1 Department of Biology, University of Aveiro, 3810-193, Aveiro, Portugal; 2 Department of Chemistry, CICECO, University of Aveiro, 3810-193, Aveiro, Portugal

## Abstract

**Background:**

Cr(V) species are formed during the intracellular reduction of Cr(VI), a ubiquitous environmental pollutant. In this study, the acute toxicity of a physiologically stable Cr(V) compound, [Cr^V^-BT]^2- ^(BT = bis(hydroxyethyl)aminotris(hydroxymethyl)methane) was investigated in the male reproductive system of sexually mature 60-day-old male ICR-CD1 mice.

**Methods:**

Eight-week-old animals were subcutaneously injected daily with a dose of *ca *8 μmol of Cr/mouse, during 5 days. The control group was injected with 0.5 mL of BT buffer. Testis and epididymis morphology was evaluated using light and transmission electron microscopy. Epididymal sperm counts, motility and acrosome integrity were also assayed using standard methods.

**Results:**

Seminiferous epithelium abnormalities were detected in the Cr^V^-BT experimental group, including intraepithelial vacuolation, and remarkable degeneration of Sertoli cells, spermatocytes and spermatids. The premature release of germ cells into the tubular lumen was also evident. Histological evaluation of epididymal compartments revealed apparently normal features. However, the epididymal epithelium presented vacuolation. [Cr^V^-BT]^2- ^induced a reduction in sperm acrosome integrity. However, sperm motility and density were not significantly affected.

**Conclusion:**

This *in vivo *study using a Cr(V) compound, provides evidence for the potential reproductive hazards caused on male reproductive system by species containing chromium in intermediate oxidation states.

## Background

Cr(VI) compounds have been declared potent occupational carcinogens by IARC [1]. Although the first report on the development of lung cancer in the chrome ore industry appeared *ca *80 years ago [2], several epidemiological studies among workers of almost one hundred different professional categories (chrome plating, stainless-steel welding, pigment and leather tanning) were published since [3-5]. Numerous and different *in vitro *and/or *in vivo *studies have been undertaken either in animals or in humans and today it is known that there is a close relationship between workers health and the amounts of industrial pollution provoked by industries manufacturing chromium containing materials [1,6,7].

Cytotoxicity and carcinogenicity of Cr(VI) depend on intracellular reduction towards stable and inert Cr(III) compounds [8,9]. During this process Cr(V) and Cr(IV) intermediate states have been gaining major relevance [10-12]. Cr(V) compounds appear as good candidates to elucidate the not yet fully understood mechanism of the intracellular trail of chromium [11,12].

Cr(V) complexes possessing α-hydroxy-carboxylate moieties are believed to act as both structural and biomimetic models for a range of Cr(V) species generated *in vivo *from Cr(VI) in intracellular media [11,12]. The [Cr^V^-BT]^2- ^complex, has a "design" which exploits an adequate co-ordination capacity to the Cr metal centre together with a strong chelate effect, and is stable under physiologically conditions over a rather long period of time, and therefore seems to be an adequate Cr(V) complex to be explored for *in vivo *studies [13].

Chromium *in vivo *studies in small rodents have dealt essentially with the effects of this element in the usual toxicological target organs. Recent works concern, for example, kidney and liver [10,14], pancreas [15], lungs [6,16-18], brain [19], male reproductive organs [20], skin [21], or even blood [22]. These studies have comprised both particulate chromium compounds, mainly related with lung pathology [16], together with soluble compounds containing chromium in all the available oxidation states, more adequate or available to reach other target organs.

In previous works we have undertaken studies on Cr(VI) and Cr(V) induced alterations on mouse liver [14], and spleen histology [23]. Also, the functional properties of Sertoli cells from mice exposed to Cr(V) were investigated [24]. The purpose of the present study was to assess the effects of [Cr^V^-BT]^2- ^on the morphology of testis and epididymis of mice.

## Methods

Bis(hydroxyethyl)aminotris(hydroxymethyl)methane, (BT) buffer (Fluka) was used and the pH of the solution was adjusted to 7.4 by addition of diluted HCl.

### Preparation of the Cr(V) compound [Cr^V^-BT]^2-^

[Cr^V^-BT]^2- ^was prepared *in situ *from a Cr(V) precursor, Na [Cr^V^O(ehba)_2_] (ehba = sodium bis(2-ethyl-2-hydroxybutanoate)oxochromato(V)), following a method described elsewhere [13].

### Animals and Treatment

The *in vivo *studies were carried out on 10 (*per *group) sexually mature 60-day-old male ICR-CD1 mice (Harlan Interfauna Iberica SA, Barcelone, Spain). Mice were housed at a constant temperature (22 +2°C), relative humidity (40–60%) *vivarium *on a light-dark 12 h/12 h cycle; water and food were provided *ad libitum*; animals were allowed to acclimatise for one week before experimental use. After this period one group of mice was subcutaneously injected daily with a dose of 8 μmol of Cr/mouse over 5 days [15]. A control group of animals was similarly injected with the vehicle (0.5 mL BT). On day six, all animals were sacrificed, under ether anaesthesia, for testis and epididymis removal.

All assays with animals were conducted in accordance with the institutional guidelines for ethics in animal experiments (Rule n° 86/609/CEE – 24/11/92).

### Ultrastructural studies

Body and organ weights were recorded. Some fragments of testis and cauda epididymis were fixed by immersion in 2.5% glutaraldehyde for 2 hours, postfixed with osmium tetroxide, and routinely prepared for transmission electron microscopy studies. Semithin sections, made with a Supernova Reichert ultramicrotome, were stained with toluidine blue for light microscopy observation. Ultrathin sections (golden colour of interference) were also prepared using a diamond knife. Then, these sections were stained with uranyl acetate and lead citrate, and observed under a Hitachi electron microscope at 100 kV.

### Sperm motility

Both epididymides were removed to a Petri dish containing Modified Tyrode's medium prepared according to Fraser [25]. The cauda epididymis was disrupted through an incision with a scissors previously wrapped with Parafilm^®^, and was kept for 30 min at 35°C to promote the release of sperm into the medium. Then, sperm were collected with a micropipette (aliquot 20 μl) to a slide for motility analysis. Sperm motility was determined by counting all progressive sperm, the non-progressive and the immotile sperm in the same field. In each preparation at least 100 sperm was counted. This motility assessment was repeated in a new preparation from the same semen sample.

### Sperm concentration

A small amount of sperm suspension was collected with a tube and was centrifuged for 5 min to 1000 g. The pellet was suspended in new media. A small amount (20 μL) of suspension was diluted 1:1 with distilled water. The sperm concentration was determined by counting the number of cells on a haemocytometer (Neubauer Improved Chamber).

### Acrosome integrity

Acrosome integrity was determined according to Lu and Shur [26]. The sperm were fixed in 5% formaldehyde in PBS for 30 min at 23°C. Fixed sperm were collected by centrifugation at 1000 g for 5 min and were then resuspended in 500 μL 0.1 M ammonium acetate pH 9.0. Sperm were washed once on the buffer and then resuspended in 100 μL ammonium acetate. An aliquot (20 μL) of this suspension was dried on a glass slide and was stained for 2 min at 23°C with 0.22 Coomassie blue G250 in 50% methanol/10% glacial acetic acid. After staining, the slides were rinsed with tap water, air-dried and mounted. The acrosome integrity was assayed by an intense staining on the anterior region of sperm head under 400× magnification.

## Results

**Figure 1:**
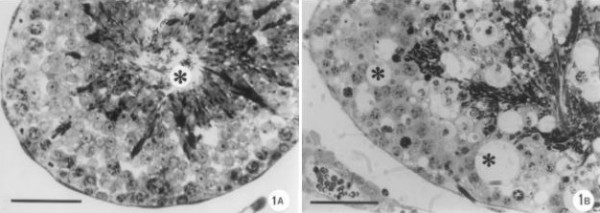
**(A-B) – Light micrographs of the testis. **Toluidine blue staining; (A) Control. Normal germinal epithelium evidencing a lumen (*); bar = 40 μm. (B) Cr(V)-treated group. Remarkable degeneration and disorganization of the seminiferous tubules is noted. Some intraepithelial vacuoles are evidenced (*). The sloughing of necrotic cells are also seen; bar = 40 μm.

In the current study, all animals survived until the end of the dosage period. Additionally, no abnormal behaviour was observed, and at sacrifice, none of the animals evidenced any macroscopic lesions. Their body weight at the start and at the end of the experiment was 32 ± 3 g, and no loss or gain in body weight was noticed along the experiments. Also, no differences were noted between the organ weights of controls and Cr(V)-injected animals over the period of these experiments (testis weight = 267 ± 2 mg; epididymis = 96 ±1 mg). The seminiferous tubules in control mice demonstrated regular features (Figure 1A). However, degenerative histological changes were observed in the epithelium of seminiferous tubules of treated animals (Figure 1B). These changes were uniform within the testis, ranging from Sertoli cells to highly mature germ cells. Intraepithelial vacuoles were clearly observed. Abnormalities affecting nearly all stages of germ cell development were seen in the seminiferous tubules, being evident necrotic mass of germ cells. In addition, all the animals injected with [Cr^V^-BT]^2- ^were affected equally.

**Figure 2:**
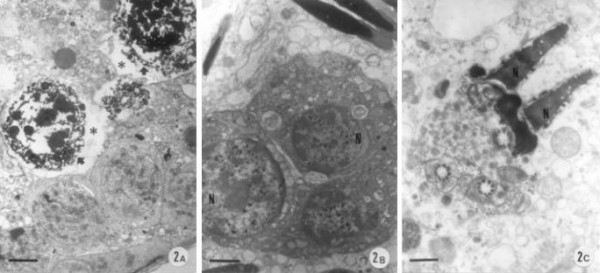
**(A-C) -Electron micrographs of the seminiferous epithelium in Cr(V)-treated group. **Uranyl acetate and lead citrate staining; (A) Ultrastructural morphology of the basal portion of a seminiferous tubule exhibiting widening of intercellular spaces (*) and necrotic germ cells (arrows); Bar = 3 μm; (B) Portion of a degenerative symplast where several nuclei (N) are seen; Bar = 2 μm; (C) Abnormal aspect of a late giant spermatid evidencing the head and the neck pieces; N – nucleus; Bar = 1 μm.

The ultrastructural features of the basal as well as the upper compartments of the seminiferous tubules in control animals revealed regular morphology. However, [Cr^V^-BT]^2- ^injected mice evidenced degenerative changes within the Sertoli cells and irregular features of spermatids, and displacement towards the lumen (Figures 2A–C). Necrotic and vacuolated germ cells were also present.

**Figure 3:**
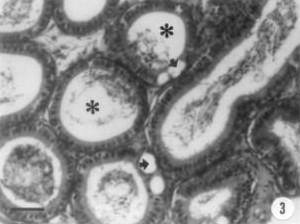
**Light micrograph of the epididymis in Cr(V)-treated group. **Some epithelial vacuoles are evidenced (arrow). Reduction of cells within the lumen is also shown (*); toluidine blue staining; Bar = 60 μm.

Epithelial cells of cauda epididymis from [Cr^V^-BT]^2- ^treated animals also exhibited a large number of vacuoles (Figure 3). However, other portions like the boundary tissue, revealed apparently normal features.

Table 1 shows that [Cr^V^-BT]^2- ^induced a slight reduction in sperm motility and density, although the differences when compared to controls were not statistically significant. Sperm acrosome integrity of mice treated with [Cr^V^-BT]^2- ^was significantly lower than sperm acrosome integrity of the controls.

**Table 1:** Effect of [Cr^V^-BT]^2- ^on sperm motility (%), density, and acrosome integrity (% of total)

		Control	[Cr^V^-BT]^2-^
Motility (%)	Progressive	34.0 ± 2.2	27.1 ± 7.2
	Non-progressive	20.3 ± 4.1	22.9 ± 7.8
	Immotile	45.8 ± 3.1	48.1 ± 7.4
Density (×10^6^)		10.1 ± 2.02	9.07 ± 2.96
Intact acrosomes (%)		82.4 ± 1.98	73.6 ± 2.2*

Note: Data are mean and standard deviation. *Values are significantly reduced (*P *< 0.05) compared to the controls, using *t*-test.

## Discussion

Cr(VI) is known to be toxic to animals and humans [3]. However implementation of both environmental and occupational regulations have been established to decrease of the hazards caused by the different types of compounds of this element. Nevertheless their associated toxicity mechanism(s) still remain unclear. For this reason, a better knowledge of the action of chromium species in intermediate oxidation states, for example, that Cr(V), is relevant in the understanding the harmful effects of chromium.

Mainly due to the poor knowledge of the role of chromium in the intermediate oxidation state Cr(V), which is considered the first step in the potentially long and harmful Cr(VI) intracellular reduction [1,3,11,12], *in vivo *experimental studies with [Cr^V^-BT]^2- ^have been undertaken in the present work.

The results of this study indicate that Cr(V), in the form of [Cr^V^-BT]^2-^, is a male reproductive toxicant, causing several histological and ultrastructural changes in mice spermatogenesis, including alterations in both Sertoli and germ cells. The presence of strongly stained exfoliated germ cells in the lumen of seminiferous tubules of [Cr^V^-BT]^2- ^treated animals demonstrates a degenerative process in this epithelium. As shown by the results of the present work, the exfoliation process is remarkable when compared with testis injuries reported for other toxicants [20,27].

Cauda epididymis also presented some epithelial vacuolation. Similar results have been described for the epididymal epithelial cells of rats treated, for example, with lead acetate [28].

These results suggest that [Cr^V^-BT]^2- ^beyond inducing alterations in testis and epididymis histology and ultrastructure also induces a reduction in sperm motility and density. Other authors have also described a reduction in epididymal sperm counts and sperm motility in rats treated with other chromium compounds, namely CrO_3 _[20]. Here, the reduction of acrosome sperm integrity in mice exposed to Cr(V) is a sign of sperm lost of fertilizing potential, reflecting its inability to penetrate the zona pellucida [29]. Our previous studies have shown that Cr(V) affected the functional properties of Sertoli cells [24]. These results show evidence that other components of testis are important toxicological targets of Cr(V) compounds.

Keeping in mind that the mammalian testis and epididymis provide a suitable and necessary environment for spermatogenesis, maturation, and storage of spermatozoa, the damaged induced by [Cr^V^-BT]^2- ^compound appears of great importance in the understanding of the biological effects of chromium in regard to male reproductive function.

Further studies are now underway in our laboratories concerning other organs and other type of experiments, which combine different factors such as several doses, time intervals, and different chromium compounds. The aim is to obtain dose-response data and eventually to identify toxicity limits of toxicology for these types of compounds and finally to clarify the mechanisms of chromium induced toxicity.

## List of abbreviations

BT – bis(hydroxyethyl)aminotris(hydroxymethyl)methane

ehba – sodium bis(2-ethyl-2-hydroxybutanoate)oxochromato(V)

CrO_3 _– Chromic acid

## Competing interests

The author(s) declare that they have no competing interests.

## Authors' contributions

MLP, TMS, and JP conceived the study, and participated in its design and coordination and helped to draft the manuscript. TMS participated in the preparation of the Cr(V) compound [Cr^V^-BT]^2-^, and completed the review of literature. RPN, and HO carried out the experimental study with mice, namely the histological, and motility, acrosome integrity, and count of sperm cells under Cr(V) exposure, and performed the statistical analysis. MLP carried out the ultrastructural studies. All authors read, discussed, and approved the final manuscript.
